# Connecting undergraduate science education with the needs of today’s graduates

**DOI:** 10.12688/f1000research.5710.1

**Published:** 2014-11-14

**Authors:** Viviane Callier, Richard H. Singiser, Nathan L. Vanderford

**Affiliations:** 1 The Ronin Institute, Montclair, New Jersey, USA; 2 Department of Natural Sciences, Clayton State University, Morrow, Georgia, USA; 3 Markey Cancer Center, University of Kentucky, Lexington, Kentucky, USA; 4 The Graduate Center for Toxicology, University of Kentucky, Lexington, Kentucky, USA

**Keywords:** undergraduate education, science curricula, work experience, job market, job skills

## Abstract

Undergraduate science programs are not providing graduates with the knowledgebase and skills they need to be successful on today’s job market. Curricular changes relevant to today’s marketplace and more opportunities for internships and work experience during students’ secondary education would facilitate a smoother transition to the working world and help employers find graduates that possess both the hard and soft skills needed in the workplace. In this article, we discuss these issues and offer solutions that would generate more marketplace-ready undergraduates.

## Introduction

The premium for an undergraduate degree is high: compared to high school graduates, college graduates in Science, Technology, Engineering and Mathematics (STEM) fields earn on average $1.5 million more over their lifetime ( Austin, 2014). This effect remains even after controlling for family background and other variables that could differentiate the population of students that pursue a college education from those who do not. Thus, attending college and studying a STEM field is still worth the cost ( Daly & Bengali, 2014) despite the ever-increasing tuition rates, the increasing burden of student debt ( Ernst, 2014), and the bad job market students encounter upon graduation ( Weissman, 2014). Notwithstanding, successfully obtaining an education certainly does not guarantee success in today’s job market ( Bersin, 2014).

Undergraduate education is badly in need of reform. Receiving an education is not the same as receiving job training, and too many students graduate with heavy debt and are ill-equipped to thrive in today’s job market ( Carpenter, 2014). The US Census Bureau has documented that many students cannot find jobs after graduation, and many of those who do find themselves employed in work that does not fully match their education/training. Students would be better served by an education that is integrated with the job market they will encounter post-graduation, and one that provides not only technical skills but also the soft skills that are most in demand by employers such as communication and interpersonal skills; decision-making skills; time and project management skills; problem-solving skills, and the ability to learn new skills quickly ( The Association Of American Colleges and Universities, 2010; The Association Of American Colleges and Universities, 2013; Tugend, 2013; White, 2013). In other words, science training at the undergraduate level should move beyond rote memorization of facts and personal character building such as persistence, perseverance, or motivation; it needs to become specific and relevant to jobs.

Most departments still use an old curriculum to teach traditional chemistry, biochemistry, biology, and molecular biology. Most students receive the same general curriculum no matter what they want as a career: find a job in industry, go to graduate school to do research, go to medical school to become a practicing physician, etc. As a consequence of undergraduate institutions doing a poor job of preparing students to be competitive for meaningful jobs upon graduation, many students pursue additional graduate training simply because they are not aware of other ways in which their undergraduate science degree could be used.

Currently, many agencies central to biochemistry and molecular biology have made curriculum recommendations. For example, the National Research Council has made some recommendations but these have not been widely implemented and miss the mark in terms of preparing highly functional, work-ready graduates, because they are too focused on traditional curricula and classroom-learning (2010). Although funding agencies, such as the National Science Foundation (NSF), push for education and outreach activities in the “broader impacts” criteria for grants, they have not sufficiently emphasized professional development of trainees specifically with respect to today’s job market. To reform undergraduate science education, we discuss below our suggestions of updating curricula and integrating work experience into programs.

## Curricular changes

At many universities, the current curricular model is outdated and employers frequently complain that graduates do not emerge with the skills they need ( Dostis, 2013). Disciplines are largely compartmentalized for historical reasons, yet most creative and innovative work comes from bridging disciplines and using concepts and tools from a variety of fields to solve important problems.

One solution is to build in interdisciplinary topics within standard STEM courses in a way that will allow students the opportunity to explore current problems in environmental science, energy fields and/or public health. For example, green/sustainable chemistry—currently a central theme in all the divisions at the Environmental Protection Agency (EPA)—could be incorporated into traditional biochemistry curriculum. Green chemistry is an interdisciplinary topic and needs to be addressed from a variety of perspectives: chemical synthesis, environmental health, and the biochemistry and molecular biology of mechanisms of action. Evidence suggests that students show great interest in the research opportunities in green chemistry and risk assessment, and students themselves clearly are pushing for incorporating current issues in energy, environment and health into their core science curriculum ( Goodman, 2009). These are excellent topics for teaching biochemistry and molecular biology students about how interdisciplinary life science topics interconnect with public health.

Current research and marketplace issues are highly interdisciplinary, and thus, students should be trained in interdisciplinary work. Another example of this is in the collaboration between mathematicians and biologists to understand metabolic systems (e.g., folate metabolism, or insulin signaling) in cells. The function of the network is an emergent property that cannot be understood at the level of individual components. The response of metabolic networks to perturbations cannot be analyzed by verbal arguments; instead, it is necessary to describe the network using a system of differential equations. This allows researchers to study its dynamic behavior with simulations. The simulations will in turn suggest interesting predictions about network function to test experimentally in the lab. The feedback between experiment and theoretical modeling is a powerful approach to complex biological problems and is only possible when interdisciplinary teams work together.

Interdisciplinary training in teams provides students the opportunity to develop soft skills such as communicating with researchers in different fields—each of which has specialized language and concepts. In addition, coursework in mathematical biology is an opportunity for STEM students to receive adequate training in quantitative skills (mathematics, statistics and data analysis) and computer programming. These skills are not only critical for pursuing a research career, but are also highly transferable skills that are valued by employers in a variety of fields.

Undergraduate programs could also take lessons from innovative graduate school initiatives. A course co-organized by the Society for Cell Biology and the Keck Graduate Institute, and funded by the biotech company EMD Millipore, Inc., provides a “crash course” for 40 selected graduate students and postdoctoral fellows interested in transitioning to careers in the biotechnology industry. The course provides MBA-style training, professional development workshops, and a team-based project. Funding from EMD Millipore is a generous investment in the training of scientists that the company may ultimately recruit. The demand for such programs is extremely high and there is clearly a need for more programs like this because STEM graduate programs currently fail to prepare their students (or postdoctoral fellows) for jobs outside of academia. Similar programs could be established in the undergraduate setting to fill a similar gap. We are aware of some institutions that are moving in this direction. For example liberal arts colleges such as Mount Holyoke, which are traditionally not focused on job-training, are creating an entrepreneurial track and developing a program focused on environmental sustainability ( Weir, 2014). Connecticut College, another liberal arts college, has created a program ( Connecticut College’s Career Enhancing Life Skills) to help undergraduates identify and develop a career path starting from their first year in college and to establish connections with potential employers throughout their undergraduate career. In addition to helping students, in almost every context, enhancing the communication between potential employers and faculty could help identify the skills that are currently lacking in many of the graduates currently produced by universities and lead to productive dialogue about curricular changes to remedy this issue.

## Work experience

In addition to incorporating curricular changes, departments and institutions should be providing bridges to the workplace such as internships. These provide critical work experience leading to the development of skills that students cannot get in the classroom, such as firm-specific technical training, but also soft skills such as working collaboratively, facilitating group decision-making, serving customers, and sales/marketing. Internships and work experience also provide critical networking opportunities that may lead to job opportunities (job offers, referrals, recommendations, etc.). Some universities, such as the Ira A. Fulton Schools of Engineering at Arizona State University, have already created partnerships with industry for mentorship and internships. Urban universities could readily incorporate internships into their programs as they have the advantage of being surrounded with companies which can offer internship experiences; rural universities could create programs with willing companies that could help students with logistics such as transportation and housing. Ultimately, how better for undergraduates to obtain the real world working experience needed to successfully gain employment after graduation than by working in the real world as part of their education?

## Conclusions

In today’s competitive job market, students need to emerge from their undergraduate STEM education with relevant technical skills as well as soft skills such as creativity, resourcefulness, intellectual curiosity, respect for others, ability to be self-directed yet able to work effectively as part of a team. Most importantly, they should emerge with a good understanding of the job options they have in a variety of sectors, work experience, and a network of professional contacts that will help them move forward in their careers with confidence, clarity and purpose.

We propose the following recommendations for changes to undergraduate STEM curriculum to better prepare students to thrive in the job market they will have to navigate upon graduation:

1) Universities/departments need to update traditional core curricula to include interdisciplinary topics that highlight connections between the standard curriculum and current, real-world STEM issues. To achieve this, there are three levels of change that institutions could invoke; these levels increase in difficulty and impact both on the institution and on students, but ultimately these changes would add significant value to students’ career development.
First, topics such as green chemistry and computational biology could be the focus of at least one lecture per semester in standard chemistry, biology/molecular biology, and/or biochemistry courses. This would be an easily change in the core curricula that would introduce students to topics and skills that directly apply to currently trending marketplace issues.
Second, STEM programs could encourage students to take non-science courses that are directly relevant to the job market. These courses could be taken as part of students’ elective coursework. We suggest that STEM programs should encourage students to take courses that would build business acumen (for example, courses on organizational behavior, leadership, entrepreneurship, strategy, and operations management); develop interdisciplinary teamwork skills through the integration of topics covering biochemistry/molecular biology, math, and computer programming/coding, public health; and lastly, enrich workplace readiness through career development topics including interviewing, resume building and networking. Universities could develop a “Preparing STEM Professionals” certificate program that would give students’ incentive to enroll in these types of courses.
A third, stretch solution, would be for institutions to create entirely new courses that address the intersection of the standard core curricula with today’s most important global topics. Some institutions are taking steps in this direction. For example, the chemistry and biochemistry courses at California State University at Fullerton include such offerings as *biotechnology: science, business, and society*; *environmental pollution and solutions*; *introduction to computational genomics*; *advanced computational biochemistry*; and *internships in chemistry and biochemistry*. Other institutions should move in similar directions. As part of these courses, students should be given opportunities to work collaboratively on projects in interdisciplinary teams, as the ability to work as part of a team is highly valued by employers. Training in quantitative data analysis and programming—sorely lacking in too many undergraduate biology/chemistry/biochemistry programs—should also be emphasized.
Ultimately, building the interdisciplinary and “soft” skills employer’s desire should be the focus on these curricular changes. The curriculum should teach students to think critically and creatively about current and future problems that need solving and that will be valued by employers.
There are likely existing programs that are achieving the outcomes we are suggesting. It would be useful for publishers to coordinate a series of articles on this subject to build awareness of the curricular changes that are already being implemented in institutions across the country and to develop guidelines and best practices for universities as they reform and update their STEM curricula to make them work-ready.
2) Universities should provide impactful opportunities and support for internships and work experience. It is through these types of experiences that students will truly gain the most useful work preparedness during their undergraduate career. Students will build real work skills and develop contacts that will be important for future employment. Perhaps the least challenging way to accomplish meaningful internships is for institutions to form formal partnerships with local or regional companies.
Many internship programs have been developed within STEM programs. For example, the Virginia Commonwealth STEM Industry Internship Program links undergraduate STEM students to paid internship positions with companies throughout Virginia; the National Homeland Security-STEM Summer Internship Program provides undergraduate juniors and seniors the opportunity to work with homeland security professionals and researchers for up to ten weeks during the summer; and the University of Connecticut’s UConn-TIP Bioscience and STEM Summer Research Intern Program pairs students with University technology start-up companies for mentored summer research internships. These are shining examples of programs that could be emulated across all undergraduate institutions.
To further incentivize integrating work experience into undergraduate curricula, we believe that funding agencies, such as NSF and the National Institutes of Health, have a key role to play. In the same way that funding agencies have promoted education and outreach in the “broader impacts” criterion for grants, they should also emphasize the need for clear, actionable career development opportunities (in academic *and non-academic* settings) for students. For example, in addition to NSF funding Research Experiences for Undergraduates (REUs) which are largely at academic institutions, NSF and NIH could also organize bridging experiences for students to explore research in industry, the world of science policy, and careers in science writing and editing. Funding agencies could develop workforce innovation funding opportunities that could incentivize the creation of unique solutions to creating work experience for undergraduates and these novel programs could serve as models for other institutions. Ultimately, funding agencies could drive a culture of creating practical work experience as part of undergraduate education.
Again, some institutions have found unique ways to successfully incorporate work experience into undergraduate STEM curricula in a way that benefits both the institution and students. Publishers could commission articles from such programs across to demonstrate their success, highlight challenges faced in development of such initiatives, and to establish discussions that may lead to the development of guidelines and best practices for undergraduate internship programs.

Given the rising costs of a college education, it is imperative that students emerge with their degrees with skills relevant to the job market. Too many employers complain that they can’t find the right talent and too many graduates are un- or under-employed. Changes in the undergraduate education system—curricular changes and integrated work experience—could remedy this problem. We encourage institutions and organizations to discuss the success and challenges they have faced in implementing such changes to the undergraduate education experience.
