# Supplementary material for: Connecting undergraduate science education with the needs of today’s graduates
Source: F1000Res. 2014 Nov 14;3:279. [Version 1] doi: 10.12688/f1000research.5710.1 (PMC4304216; doi:10.12688/f1000research.5710.1)
Supplement: Peer review history

# Peer review: Referee response for version 1

**DOI:** 10.5256/f1000research.6105.r6827

**Published:** 2014-12-18

**Authors:** Marie-Claire Shanahan

**Affiliations:** 1 Werklund School of Education, University of Calgary, Calgary, AB, Canada

The authors have hit on a very important issue here, and I am really pleased to see ongoing discussions of how undergraduate science education can be better tailored to students’ needs. The authors also introduce interesting and relevant examples of integrated curriculum topics (such as green chemistry) and of successful STEM career programs (such as Connecticut College’s Career Enhancing Life Skills).

Given the importance of these issues, I would really encourage the authors to look at how the argument might be strengthened, in particular with support from empirical and peer-reviewed sources. The authors are clear in their views in a way that is appropriate for an opinion piece, but the factual claims that are made in service of the overall argument need better supporting sources. I understand that this is an opinion piece and am not suggesting an exhaustive review of the literature, just attention to a few key findings related to the arguments that are made here. For example, some claims are supported with weak evidence from opinion articles from media sources rather than empirical and peer-reviewed sources. E.g., “Too many students graduate with heavy debt and are ill-equipped to thrive in today’s job market” (p. 2) citing only Carpentier (2014), an opinion piece from the NYT that is supported with only an online survey from a job search website. Similarly the claim about the substance of employers' complaints about graduates (p. 2) is supported with a brief news article about a poll commissioned by an online homework help website. It would be important to at least examine the full report to ensure that the data is appropriate for making claims in an academic opinion piece. 

Other claims are made with no support at all, e.g., “Many students pursue additional graduate training simply because they are not aware of other ways in which their undergraduate science degree could be used.” (p. 2). A claim that quantitative skills are an example of “highly transferable skills that are valued by employers” is also unsupported. Off hand I don't know of any studies that specifically address quantitative skills as valued by employers but it could fall within the mismatch that Hernández‐March *et al *(2009) find in field-specific practical skills that employers desire but perceive that students lack. Sagen, Dallam and Laverty (2000) also find that quantitative training is related to job search success for undergraduates though they do not examine employers' desires directly.

The paragraph that spans p. 2-3 describes several good examples such as Mount Holyoke and Keck Graduate Institute. It is a good illustration of beginning share examples and best practices, as the authors advocate in their conclusion. It could, again, be stronger if there were connections to some of the published case studies that try to assess claims like these in relation to specific programs, e.g., Junge *et al* (2010). Links to published case studies would also be very valuable in supporting the suggestion that publishers offer more venues for sharing best practices, challenges and successes. It would be important to acknowledge the venues that do exist, while also advocating for more.

Overall, I commend the authors on tackling a very important issue and encourage their efforts to push this discussion forward. I think that their argument could be greatly strengthened, however, with better attention to at least a few key pieces of the literature in the area of workplace and employability programs in undergraduate education.  They would find good support for their overall aims but also be able to make more nuanced arguments about how the important goal of improving undergraduate science education can be accomplished. 

To that end, here are a few pieces that might be of interest to the authors:

Cranmer, S. (2006). Enhancing graduate employability: best intentions and mixed outcomes. *Studies in Higher Education*, *31*(2), 169-184. 

A study of university departments examining their faculty members’ practices for teaching employability skills with attention to how well their goals are achieved. 

Coll, R. K., & Zegwaard, K. E. (2006). Perceptions of desirable graduate competencies for science and technology new graduates. *Research in Science & Technological Education*, *24*(1), 29-58.

Study of various stakeholders (including both employers and faculty) on what skills and competencies they prioritize, with a specific focus on “work-integrated learning”. 

Hernández‐March, J., Martín del Peso, M., & Leguey, S. (2009). Graduates’ skills and higher education: The employers’ perspective. *Tertiary Education and Management*, *15*(1), 1-16.

A large study of Spanish HR directors and company managers to examine what they see as required skills and what mismatches they perceive between undergraduate training and job requirements. 

Burke, V., Jones, I., & Doherty, M. (2005). Analysing student perceptions of transferable skills via undergraduate degree programmes. *Active Learning in Higher Education*, *6*(2), 132-144.

This is a case study of students at one undergraduate institution, examining their perceptions of the skills they have developed during their degree programs and how confident they are in their abilities to transfer those skills to a workplace environment. 

Sagen, H. B., Dallam, J. W., & Laverty, J. R. (2000). Effects of career preparation experiences on the initial employment success of college graduates. *Research in Higher Education*, *41*(6), 753-767.

A large study looking the factors that predict employment success of college graduates one month after graduation. Their regression model looked at a wide variety of factors from internship experiences to personal characteristics.

Junge, B., Quiñones, C., Kakietek, J., Teodorescu, D., & Marsteller, P. (2010). Promoting undergraduate interest, preparedness, and professional pursuit in the sciences: an outcomes evaluation of the SURE program at Emory University. *CBE-Life Sciences Education*, *9*(2), 119-132.

A long term evaluation study of a summer research program that aimed to increase student preparedness for both graduate school and industry.

I have read this submission. I believe that I have an appropriate level of expertise to confirm that it is of an acceptable scientific standard, however I have significant reservations, as outlined above.

# Response

**Published:** 2015-01-21

**Authors:** Nathan Vanderford

**Affiliations:** University of Kentucky, USA

Dear Dr. Shanahan,

We thank you for reviewing our article and for your comments.  You clearly have a detailed understanding of the literature focusing on these issues. We have given your critique a great deal of thought, and we have decided to forgo submitting a revision to our article based on your comments primarily given the fact that *F1000Research* reviews can be independently cited. Ultimately, we feel that a revised version of the article would add no additional value beyond what is already captured in your critique. We therefore encourage readers to refer to and authors of subsequent work to reference your referee report. 

Thank you again for your review.  

Viviane Callier, Richard H. Singiser, Nathan L. Vanderford

# Peer review: Referee response for version 1

**DOI:** 10.5256/f1000research.6105.r6825

**Published:** 2014-12-03

**Authors:** Laurence Lurio

**Affiliations:** 1 Department of Physics, Northern Illinois University, DeKalb, IL, USA

This is more of an opinion piece than a research article. The idea of incorporating practical applications into STEM education is obviously a good idea, but the obstacles to implementation have not really been addressed, which is crucial.  Statements such as "science training at the undergraduate level should move beyond rote memorization of facts" seem rather naive.  No one, for a long time, has argued that undergraduate education should be rote memorization.

I have read this submission. I believe that I have an appropriate level of expertise to confirm that it is of an acceptable scientific standard.

# Response

**Published:** 2014-12-16

**Authors:** Nathan Vanderford

**Affiliations:** University of Kentucky, USA

Dear Dr. Lurio,

We thank you for reviewing our article and for your comments.  As you note, the article does contain a few statements that could, arguably, be controversial, and as an opinion article, we feel that we are warranted in expressing our views on the current state of undergraduate education and on how we see ways to improve its future state. We agree with your point that “no one, for a long time, has argued that undergraduate education should be rote memorization” yet too often that is still what we see in the classroom, and it remains a problem. We also agree that there will be challenges/obstacles to implementing our recommendations and we believe that these may vary widely from institution to institution. We hope that by specifically mentioning in the article that publishers should help commission articles from programs that are implementing practical applications – such as updated curricula and the integration of work experience – that such articles would address associated challenges/obstacles, best practices, and success stories. As such, we believe that future articles will best address your point.

Thank you again for your review.  

Viviane Callier, Richard H. Singiser, Nathan L. Vanderford

# Peer review: Referee response for version 1

**DOI:** 10.5256/f1000research.6105.r6714

**Published:** 2014-11-17

**Authors:** Genevieve Newton

**Affiliations:** 1 Human Health and Nutritional Sciences, University of Guelph, Guelph, ON, Canada

This is an interesting and timely article that presents viable solutions to some of the challenges being faced by higher education. The two main solutions presented - increasing the interdisciplinarity of STEM undergraduate curricula and providing more work experiences for students - are consistent with accepted high impact educational practices. The paper is quite brief, but nonetheless presents several concrete examples, and the authors rightfully encourage educators to share what they are doing in order to develop guidelines and best practices.

In terms of the interdisciplinary curriculum argument, I would encourage the authors to broaden this even further to include disciplines such as sociology, political science, and psychology, as many of today's scientific issues (such as climate change and the challenge of feeding the planet) can be addressed from a multitude of different angles. 

In terms of the suggestion of increasing work experience for students, it would be helpful to see more discussion of the different approaches that can be taken in this regard. For example, consideration of co-operative programs, internships, externships, and course-embedded community engaged learning projects.

Overall, this paper achieves the stated objective of describing strategies to connect undergraduate science education with the needs of today's graduates, and should prove informative to educators in STEM fields.

I have read this submission. I believe that I have an appropriate level of expertise to confirm that it is of an acceptable scientific standard.

# Response

**Published:** 2014-12-16

**Authors:** Nathan Vanderford

**Affiliations:** University of Kentucky, USA

Dear Dr. Newton,

We thank you for reviewing our article and for your comments. We agree that a number of interdisciplinary topics could (and should) be integrated into STEM curricula. We have limited the scope of our article to a detailed discussion of a few example topics and, via our suggestion within the article that others should report on their programs that have novel curricula, we hope to hear a variety of other examples that integrate a wide range of topics/disciplines into STEM curricula. This is also the case regarding your comment on additional methods for integrating work experience into STEM programs; we hope that our specific call for others to report on their programs leads to a number of other articles that share specifics about how institutions are integrating work experience into STEM curricula through a number of different methods including co-operative programs, inter/externships, etc. As such, we look forward to subsequent articles that can further address your comments.

Thank you again for your time and comments.

Viviane Callier, Richard H. Singiser, Nathan L. Vanderford
